# Photosynthetic pigments and peroxidase activity of *Lepidium sativum* L. during assisted Hg phytoextraction

**DOI:** 10.1007/s11356-017-8951-3

**Published:** 2017-04-06

**Authors:** Beata Smolinska, Joanna Leszczynska

**Affiliations:** Department of Biotechnology and Food Sciences, Institute of General Food Chemistry, Lodz University of Technology, 4/10 Stefanowskiego Str, 90-924 Lodz, Poland

**Keywords:** *Lepidium sativum* L., Hg, Chlorophylls, Pheophytins, Carotenoids, Peroxidase, Phytoextraction

## Abstract

The study was conducted to evaluate metabolic answer of *Lepidium sativum* L. on Hg, compost, and citric acid during assisted phytoextraction. The chlorophyll *a* and *b* contents, total carotenoids, and activity of peroxidase were determined in plants exposed to Hg and soil amendments. Hg accumulation in plant shoots was also investigated. The pot experiments were provided in soil artificially contaminated by Hg and/or supplemented with compost and citric acid. Hg concentration in plant shoots and soil substrates was determined by cold vapor atomic absorption spectroscopy (CV-AAS) method after acid mineralization. The plant photosynthetic pigments and peroxidase activity were measured by standard spectrophotometric methods. The study shows that *L. sativum* L. accumulated Hg in its aerial tissues. An increase in Hg accumulation was noticed when soil was supplemented with compost and citric acid. Increasing Hg concentration in plant shoots was correlated with enhanced activation of peroxidase activity and changes in total carotenoid concentration. Combined use of compost and citric acid also decreased the chlorophyll *a* and *b* contents in plant leaves. Presented study reveals that *L. sativum* L. is capable of tolerating Hg and its use during phytoextraction assisted by combined use of compost and citric acid lead to decreasing soil contamination by Hg.

## Introduction

*Lepidium sativum* L. is a fast-growing annual herb belonging to the *Brassicaceae* family. It is often cultivated in many parts of the world (Egypt, Asia, Europe, USA) for various culinary and medicinal uses. This plant is abundant in macroelement and microelement, as well as in vitamins (Nehdi et al. 2012). The main character of *L. sativum* L. is that it can grow in any type of climate and soil condition and can be sown and harvested several times throughout the year (Wadhwa et al. 2012; Nehdi et al. 2012). Its high sensitivity to phytotoxic substances makes it suitable for biological tests conducted to assess the state of soil and water environment (Janecka and Fijalkowski 2008).

*L. sativum* L. grown on polluted soil showed ability for Cd, Se, and Hg accumulation (Gill and Tuteja 2010; Elguera et al. 2013; Smolinska 2015). This creates the possibility to use of this plant in phytoremediation processes. Phytoremediation techniques are considered to be environmentally friendly. In contrast to physical and chemical methods that are already being used to clean up the soil from heavy metals, phytoremediation is low cost, less disruptive, and effective in contaminant reduction (Tangahu et al. 2011). Phytoextraction, as one of the phytoremediation techniques, consists on using higher plants to take up heavy metals from contaminated medium and accumulate them in harvestable tissues (Lasat 2002). The pollutants can then be removed by harvesting (Wang et al. 2012). The major advantages of this method are low environmental impacts, easily operated, and can be applied at a large scale (Wang et al. 2012). However, this method has also limitations. One of them is the process efficiency, which is dependent on soil and plant factors. The plant biomass and heavy metal concentration in aerial parts of plants are the key factors that decide about the phytoextraction efficiency. Application of chemical compounds can increase the effectiveness of the process (Meers et al. 2005). The role of these substances is to desorb metals from soil matrix into the soil solution, thereby facilitating metal transport into the xylem and increasing the translocation from below to aboveground parts of plants (Gao et al. 2012).

Recently, several studies have focused on using synthetic chelators to improve the efficiency of phytoextraction. Various chemical substances, such as ethylenediaminetetraacetic acid (EDTA), hydrochloric acid, thiosulfate, or iodide, have been mostly commonly employed in enhanced process (Rodriguez et al. 2016; Lomonte et al. 2011). However, the use of mentioned chelating agents has increased the concern of Hg leaching and transferring to groundwater. Therefore, other substances have being tested for assisted phytoextraction.

Citric acid (CA) as easily biodegradable and low phytotoxic compound has been suggested for chemically assisted phytoextraction of Cd- and Pb-contaminated soil (Gao et al. 2012). The previous investigations showed that CA enhanced the Hg accumulation by *L. sativum* L. (Smolinska and Krol 2012). However, its application also increased the Hg leaching (Smolinska and Krol 2012). Another studies indicated that using compost from green wastes increased Hg phytoextraction efficiency and at the same time definitely decreased Hg leaching (Smolinska 2015). Therefore, in this study, the combined use of CA and compost from green wastes was tested in assisted phytoextraction of Hg-contaminated soil.

The physiological and biochemical mechanisms involved in Hg accumulation during assisted phytoextraction are not completely understood. Hg phytotoxicity is connected with generation of reactive oxygen species (ROS), like hydrogen peroxide, superoxide radical, and hydroxyl radical (Wang et al. 2012). Accumulation of ROS in plant cells leads to disturbance of plant growth, photosynthesis, and biochemical processes. Some studies indicate that Hg treatment affected the amount of photosynthetic pigments, like chlorophylls and carotenoids (Lenti et al. 2002; Puzon et al. 2014). Hg is known to interfere with chlorophyll synthesis through direct inhibition of enzymes involved in this process (Van Assche and Clijsters 1990). As the result, the reduced chlorophyll levels have been observed in plants exposed to Hg (Moreno-Jimenez et al. 2007). Moreover, under stress conditions, part of chlorophylls might be converted to pheophytins. Pheophytins are compounds formed during the chlorophyll degradation by the losses of magnesium ions from chlorophyll (Sanmartin et al. 2011). The concomitant pheophytin accumulation and oxidative stress have been observed in plants exposed to toxic concentrations of trace elements (Gomes et al. 2016; Mobin and Khan 2007). Monitoring the changes in photosynthetic pigments in response to metal stress indicates the damage to the photosynthetic apparatus and accompanying changes in photosynthetic capacity of plant (MacFarlane and Burchett 2001). The changes in physiological processes of plant (e.g., photosynthesis) are also correlated with the activity of peroxidase enzyme. Peroxidases (PODs) catalyze the reduction of hydrogen peroxide by transferring electrons to various donor molecules such as phenolic compounds, lignin precursors, or secondary metabolites (Kim et al. 2010). Increased POD activity in response to heavy metal stress may play a key role in the cellular defense mechanism against metal toxicity (Van Assche and Clijsters 1990).

To control the level of ROS, plant cells possess an antioxidant system, composed not only from the enzymes like POD but also from metabolites like glutathione (GSH) (Sobrino-Plata et al. 2014b). GSH may play a dual protective role in plant toxic metal tolerance, both as an antioxidant and as a precursor of phytochelatins (PCs) (Sobrino-Plata et al. 2014a). PCs are synthesized from GSH and homologous biothiols by the enzyme phytochelatins synthase and it is thought that PC-metal complexes are transported to the vacuole in the final step of metal detoxification (Carrasco-Gil et al. 2011; Sobrino-Plata et al. 2014b). According to Sobrino-Plata et al. (2014a), an important mechanism of Hg detoxification is based on the high affinity for sulfhydryl groups through the formation of Hg-phytochelatin (Hg-PC) complexes.

Although the Hg phytotoxicity to higher plants has been investigated (Patra and Sharma 2000), there is lack of information about *L. sativum* L. metabolic answer to Hg and other substances, like CA and compost, used in assisted phytoextraction. Thus, the aim of the study was (1) to determine Hg accumulation in shoots of *L. sativum* L. during phytoextraction assisted by combined use of CA and compost and (2) to investigate the effects of Hg and combined use of CA and compost on chlorophylls (*a* and *b*) and pheophytins (*a* and *b*), carotenoids, and POD activity in plant shoots.

## Materials and methods

### Pot experiments

*L. sativum* L. seeds (Grono, Poland) were used in the experiments. Plants were grown in the plastic pots under optimum greenhouse conditions as described previously by Smolinska and Leszczynska (2015). Plant cultivation was provided for 7 days after sowing. During the cultivation, plants were watered with deionized water to keep soil humidity at 35%. After cultivation, the aboveground parts of plants were harvested, divided into leaves and stems, and subjected to further analysis.

**Fig. 1:**
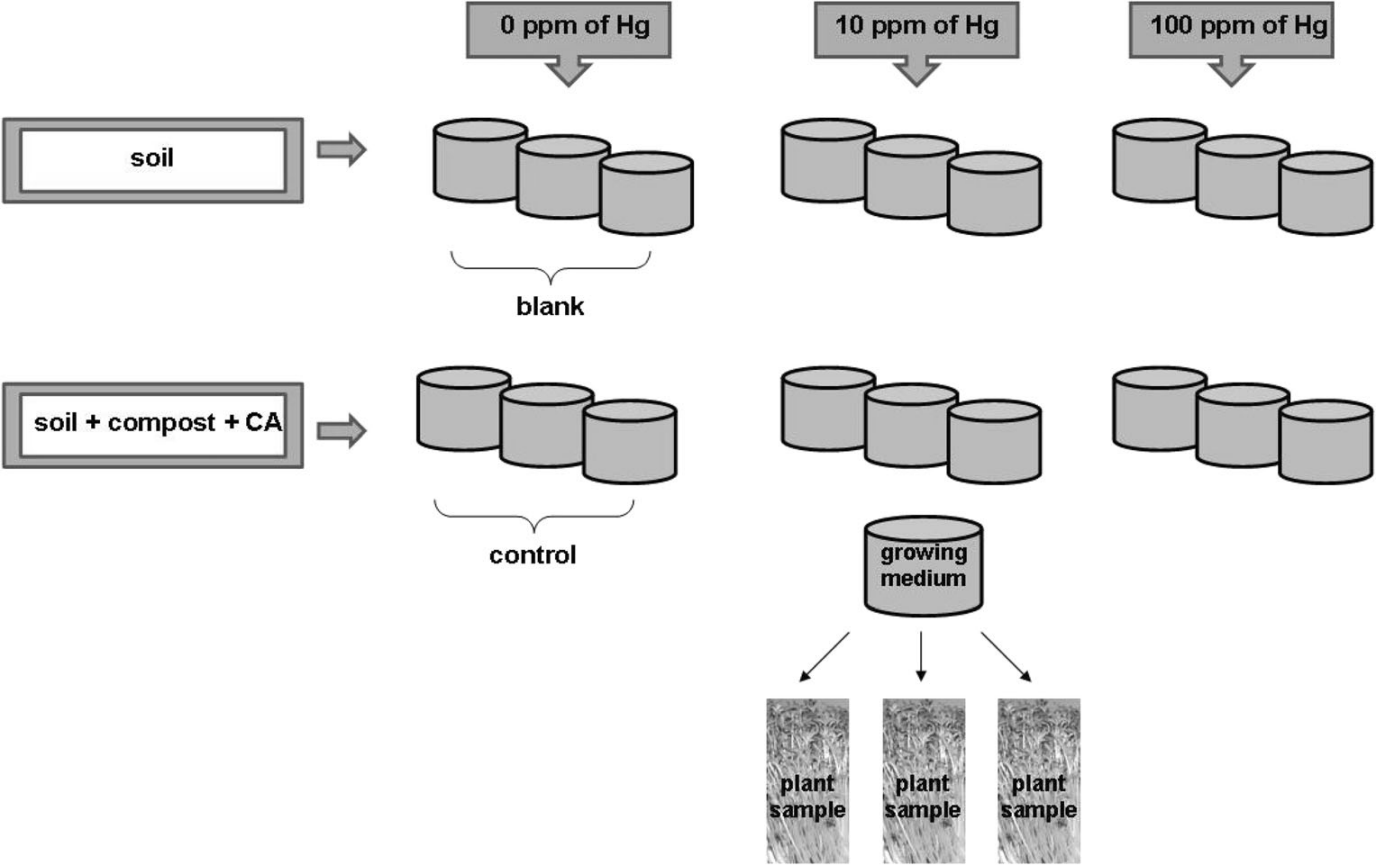
The scheme of preparation the pot experiment

All experiments were provided in greenhouse conditions with *L. sativum* L. plants. The cultivations were provided in soil or soil incorporated with compost from green wastes in soil/compost ratio 3/1. Additionally, some pots were supplemented with CA in concentration 100 mg kg^−1^ dry mass. The detail chemical characterization of both soil and compost used in experiments were presented by Smolinska (2015). Mercury (II) chloride (HgCl_2_) in concentrations 10 or 100 mg kg^−1^ dry mass was used for soil pollution. Soil samples supplemented with compost, CA, and/or Hg were left for stabilization for 7 days before seed sowing. The total experiment consists of the following six variants: one blank sample (untreated soil sample, without Hg), control sample (soil + compost + CA, unsupplemented with Hg), two soil samples treated with Hg in concentration 10 or 100 mg kg^−1^ dry mass, respectively, and two samples of soil + compost+ CA treated by 10 or 100 mg kg^−1^ dry mass of Hg, respectively. Each of pot experiment was provided in three replicates. The three of representative samples from each treatment were used for further determinations. The diagram with the preparation of pot experiments is presented in Fig. 1.

### Hg analysis

Hg concentration was determined in both soil and plant samples. Total concentration of Hg in soil was determined in air-dried samples in accordance to procedure described in details by Smolinska (2015). The soil samples were subjected firstly to acid mineralization, and then, the total Hg concentration was determined by cold vapor atomic absorption spectroscopy (CV-AAS) method. Hg concentration in *L. sativum* L. stems and leaves was determined using procedure described by Cavallini et al. (1995). Harvested aerial parts of plant were washed with deionized water to remove soil particle and dried at 35 °C to dry mass. Then, air-dried aerial parts of plants were grounded into powder and in this form were subjected to acid mineralization. Concentration of Hg was determined using CV-AAS.

Determination of Hg concentration in aboveground parts of *L. sativum* L. and soil were used for calculation bioconcentration factor (BCF) in accordance to equation presented by Zhuang et al. (2007).

### Photosynthetic pigments

Chlorophyll *a* (chl *a*), chlorophyll *b* (chl *b*), and total carotenoids (carotenes and xanthopylls) were determined spectrophotometrically in accordance to extraction procedure described by Frooq et al. (2016). Topmost fully expanded leaves were taken to extracts the pigments. Of the aqueous acetone, 85% were used for photosynthetic pigment extraction from 10 mg of fresh leaf disks. The extinction was evaluated against a blank of a pure 85% acetone at wavelengths of 663, 645, and 452.5 nm for chl *a*, chl *b*, and total carotenoids, respectively. Concentration of chl *a*, chl *b*, and total carotenoids were calculated by using adjusted extinction coefficient and equations (Lichtenthaler 1987). The chl pheophytination was estimated spectrophotometrically on the basis of pheophytin *a* (phe *a*) and pheophytin *b* (phe *b*) formation after sample acidification with 10 dot plates of 1 M of HCl solution in accordance to procedure described by Vernon (1960). All obtained values for chl *a*, chl *b*, total carotenoids, and phe *a* and phe *b* were normalized for fresh biomass.

### Peroxidase activity

One gram of aboveground part of *L. sativum* L. was grounded and homogenized in potassium phosphate buffer (50 mM, pH = 7.0), containing 0.2 mM EDTA and polyvinylpyrrolidone (PVP). The samples were centrifuged at 10,000×*g* for 10 min at 4 °C. The supernatants were used for determination of enzyme activity. The peroxidase activity POD (EC.1.11.1.7) was determined by monitoring the increase in absorbance at 470 nm during the oxidation of guaiacol (Hemeda and Klein 1990). The activity of POD was defined as 1 μmol of guaiacol oxidized during 1 min at 25 °C. POD activity was expressed in enzyme unit mg^−1^ protein. Protein content of the extracts was determined by the method of Bradford (1976).

### Statistics

Experiments were statistically analyzed using Statistica 12.0 (StatSoft Inc. 2011). Differences between treatments were evaluated with a non-parametric analysis of variance on ranks—Kruskal-Wallis test. Non-parametric approach was chosen since the normal distribution of data and homogeneity of treatment variances were not met. Pairwise comparisons between different treatments were provided using the Mann-Whitney *U* test with correlation factor of Spearman’s rank. Significance was accepted for *p* < 0.05. Data for each experiment were analyzed separately. Results presented are representative of each group of experiment.

## Results

### *L. sativum* L. growth

*L. sativum* L. plant showed its sensitivity to Hg and compost + CA during assisted phytoextraction. The plant growth was dependent on the Hg concentration in soil. Increasing Hg amount in soil caused the decrease of shoot biomass. In accordance to Table 1, the shoot biomass decreased 12 and 20% for soil polluted by 10 and 100 mg kg^−1^ of Hg, respectively, when compared to cultivation provided in unpolluted soil (blank sample). Plant exposition to Hg resulted also in disturbance in water absorption, which is described by the dry mass to fresh mass ratio (DM/FM). As it can be seen in Table 1, the DM/FM increased for both leaves and stems of *L. sativum* L. exposed to Hg.

**Table 1:** Shoot biomass of *L. sativum* L. and dry mass/fresh mass ratio (DM/FM) of leaves and stems cultivated in different variants of assisted phytoextraction (mean of nine replicates ± standard deviation (*n* = 9) ± SD)

Type of cultivation	Shoot biomass (g FM)	Leaves (DM/FM)	Stems (DM/FM)
Blank	18.89 ± 0.08	0.164	0.164
Control	19.97 ± 0.12	0.165	0.166
Hg 10	16.63 ± 0.14	0.189	0.173
Hg 10 + compost + CA	19.24 ± 0.10	0.162	0.162
Hg 100	15.14 ± 0.07	0.222	0.178
Hg 100 + compost + CA	17.04 ± 0.19	0.172	0.171

Incorporation of compost + CA to soil led to decrease the toxic effect of Hg to plant (Table 1). Plant cultivation in polluted soil substrate supplemented by compost + CA increased the shoots biomass over 15 and 12% for soil polluted by 10 and 100 mg kg^−1^ of Hg, respectively, when compared to the shoot biomass harvested after cultivation in polluted soil.

### Effect of soil amendments on photosynthetic pigments and POD activity

**Fig. 2:**
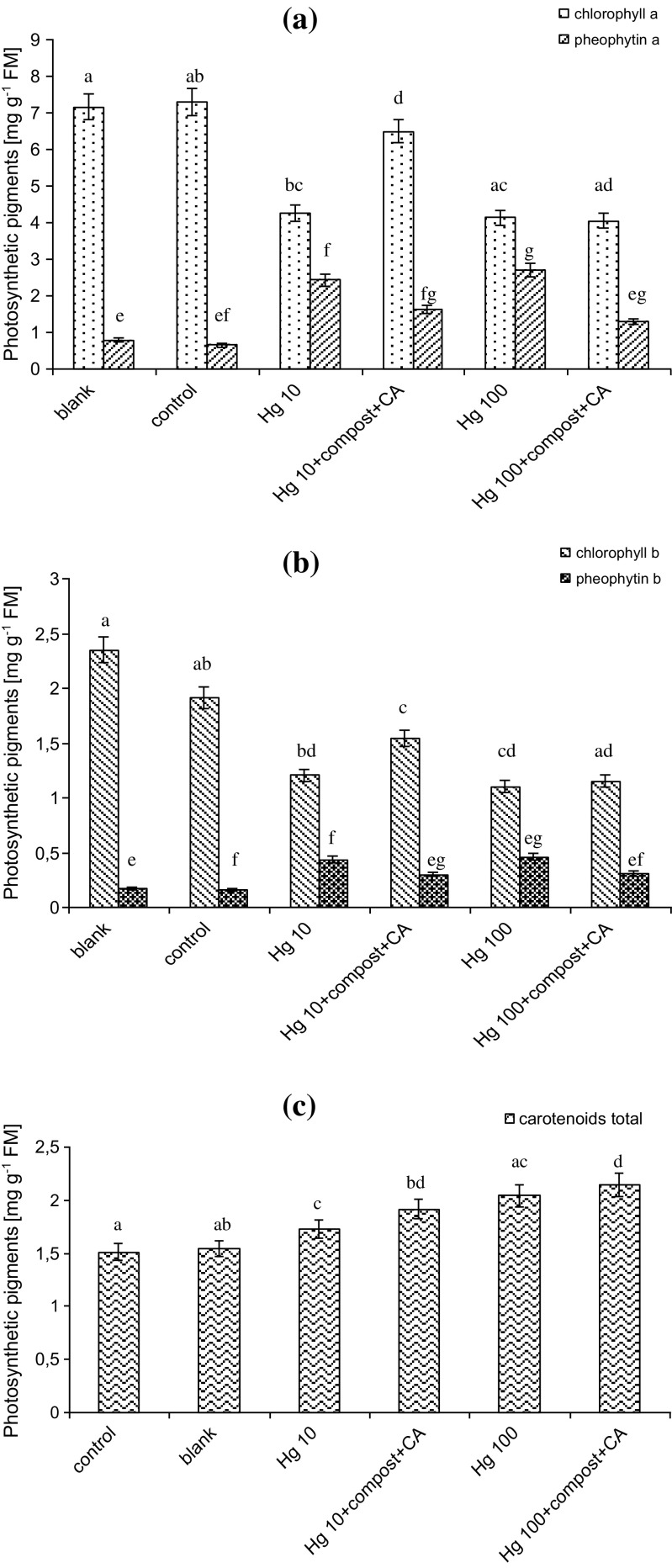
Photosynthetic pigment concentrations in leaves of *L. sativum* L. exposed to Hg and additional treatments. **a** Chlorophyll *a* and pheophytin *a*. **b** Chlorophyll *b* and pheophytin *b*. **c** Total carotenoids. *Vertical bars* represent the standard deviation of the mean (*n* = 9). *Letters* indicate that mean values are statistically different between the treatment (*p* < 0.05)

The exposure of *L. sativum* L. to Hg resulted in reduction of chl *a* and *b* in plant leaves (Fig. 2a, b). The chl reduction was Hg concentration dependent. For plant exposition to Hg in concentration 10 mg kg^−1^, the chl *a* and *b* decreased over 40 and 48%, respectively, in relation to blank sample. The reduction over 42 and 53% of chl *a* and *b* was observed after plant exposition to Hg in concentration 100 mg kg^−1^. The analysis of plant leaves for phe *a* formation showed the opposite tendency. Regarding the results presented in Fig. 2a, the highest increase of phe *a* amount occurred in plant leaves exposed to Hg ions. For soil pollution by 10 and 100 mg kg^−1^ of Hg, the concentration of phe *a* constituted over 57 and 65% of chl *a* amount, respectively. Similarly, the concentration of phe *b* in *L. sativum* L. leaves increased in the order of increasing concentration of Hg in soil (Fig. 2b). Application of compost + CA to soil polluted by Hg influenced the plant photosynthetic pigments. Although the decrease in chl *a* and chl *b* was still noticed, the reduction was significantly limited when compared with respective Hg-treated plants without compost + CA addition. In these variants of experiment, the moderate increases of both phe *a* and phe *b* concentrations were observed.

The comparative analysis of total carotenoid concentration in plant leaves exposed to Hg ions showed their increasing tendency. According to Fig. 2c, total carotenoid content changed in *L. sativum* L. plants cultivated in soil polluted by Hg in concentrations 10 and 100 mg kg^−1^, relative to blank sample. Soil amendments also led to change of total carotenoid concentrations in plant tissues. Over 11 and 5% of total carotenoid increase were observed for plant cultivated in soil substrates supplemented by compost + CA treated with Hg in relation to respective Hg-treated plants without amendments.

**Fig. 3:**
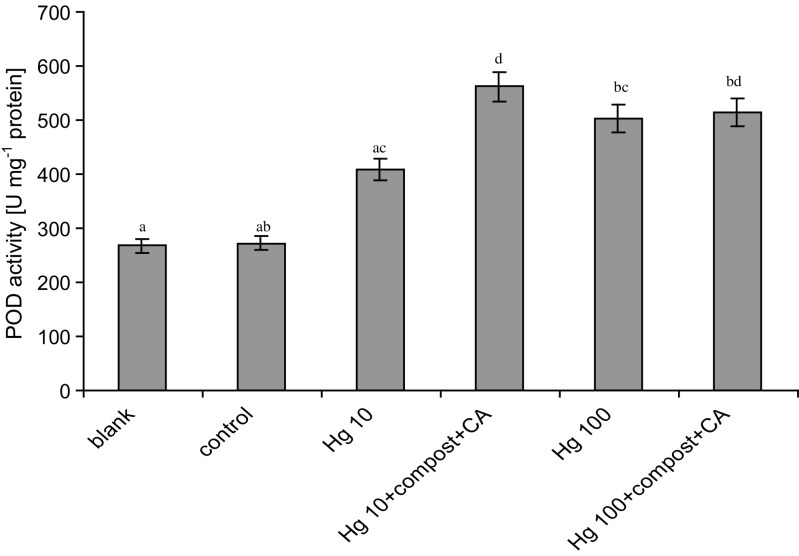
Effect of Hg on the activity POD in aerial tissues of *L. sativum* L. *Vertical bars* represent the standard deviation of the mean (*n* = 9). *Letters* indicate that mean values are significantly different between the treatment (*p* < 0.05)

The other tested plant parameter is POD activity. In accordance with Fig. 3, POD activity increased with increasing Hg concentration in soil. The POD activity was found to increase over 52 and 88% for Hg-treated plants in concentrations 10 and 100 mg kg^−1^, respectively, when compared to untreated plant samples. Moreover, the linear relationship was observed between Hg accumulation and POD activity. Combined use of compost + CA significantly enhanced POD activity in shoots of *L. sativum* L.

### Hg accumulation and assessment of assisted phytoextraction

**Fig. 4:**
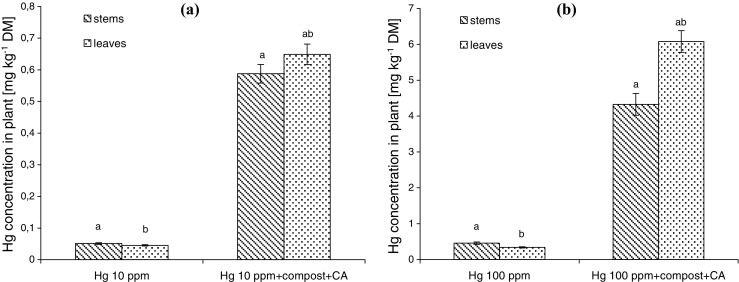
Hg concentration in aboveground parts of *L. sativum* L. exposed to Hg in concentrations of **a** 10 mg kg^−1^ DM and **b** 100 mg kg^−1^ DM. *Different letters* indicate the significant difference between the treatments (*p* < 0.05)

Hg accumulation in leaves and stems of *L. sativum* L. is presented in Fig. 4a, b. Hg concentration in aboveground parts of plant was low for processes provided in polluted soil, regardless the degree of soil contamination. Even less than 1% of soil Hg was accumulated in aerial tissues of *L. sativum* L. Soil incorporation with compost + CA significantly increased Hg concentration in plant shoots. Aerial plant tissues accumulated over 12 and 10% of total Hg for pollution by 10 and 100 mg kg^−1^ of Hg, respectively. Increased Hg accumulation was observed in both stems and leaves of *L. sativum* L. cultivated in soil with compost + CA, regardless the soil contamination. Moreover, Hg accumulation was higher in plant leaves than stems.

The ability of Hg accumulation in shoots of *L. sativum* L. was determined. The BCF values are presented in Table 2. BCF was low for plants exposed to Hg ions. Over tenfold increase of BCF was noticed for *L. sativum* L. cultivated in soil supplemented by compost + CA, regardless the degree of soil contamination. These findings suggest that *L. sativum* L. can be used during assisted phytoextraction.

**Table 2:** Bioconcentration factor (BCF) of *L. sativum* L. during phytoextraction assisted by combined use of compost and CA

	Hg 10		Hg 100	
	Soil	Soil + compost + CA	Soil	Soil + compost + CA
BCF	0.010	0.125	0.08	0.106

## Discussion

*L. sativum* L. growth was affected by Hg. The aboveground plant biomass reduction was proportional to Hg treatments. The higher Hg concentration in soil led to decrease plant biomass. The reduction of plant biomass caused by Hg was also observed for other plant species, like *Triticum aestivum* and *Cucumis sativum* (Sahu et al. 2012; Cargnelutti et al. 2006). These studies indicated that Hg in higher concentrations is highly phytotoxic to plant cells and affects the plant growth. Reduced plant growth was also indicated by increasing DM/FM ratio. In our study, Hg treatments led to increase this parameter. An increased DM/FM ratio may be explained by reduced water uptake, which in turn causes the inhibition of plant cell elongation and enlargement (Sahu et al. 2012).

*L. sativum* L. growth was dependent on the soil substrate used for plant cultivation. Incorporation of compost + CA to polluted soil caused the shoot biomass increase when compared to the Hg treatments without compost + CA application. Compost as a natural fertilizer is rich in macroelement and microelement. Furthermore, it is characterized by high organic matter content. All of these enhance the nutrition supply of plants. On the other hand, some studies indicate that CA enhanced the plant growth (Ehsan et al. 2014). It was demonstrated that CA addition increased the plant growth and biomass in *Brassica napus* L. exposed to Cd (Ehsan et al. 2014). CA is a natural organic acid that usually chelates and solubilizes essential nutrients in soil solution (Nascimento 2006). Combined use of compost + CA increased the plant biomass probably due to increased nutrient uptake. Moreover, the water uptake was not affected in plants cultivated in soil substrate supplemented by compost + CA as evidenced by DM/FM ratio.

In this study, the influence of Hg and other treatments on *L. sativum* L. photosynthetic pigments were investigated. The obtained results showed chl *a* and chl *b* decrease in plant leaves exposed to Hg. These findings are in line with investigations carried out by Puzon et al. (2014), who reported that chl *a* and chl *b* contents decreased in mature leaves of *Eichhornia crassipes* after exposition to Hg. Chen et al. (2017) also noticed the declines in photosynthetic pigments, including chl *a* and chl *b* in rice (*Oryza sativa* L.) under Hg exposure. Reduction of chl *a* and *b* in plant leaves may be explained by substitution of metal ions in photosynthetic pigments by mercuric ions (Zhou et al. 2007; Chen et al. 2017). Furthermore, in accordance to Puzon et al. (2014), the reduction of chl levels in leaves could be also attributed to the possible degradation of chloroplasts, and in turn the inhibition of chl biosynthesis which affects the physiological state, eventually leading to leaf senescence. The decrease of chl *a* and *b* in plant leaves may also affect the photosynthesis performance which reduce the plant growth (Ehsan et al. 2014).

The study showed that chl *a* and chl *b* contents drastically decreased by exposure to Hg ions. To determine if these decreases of chl were due to its degradation, the pheophytin concentrations in *L. sativum* L. leaves were investigated. In accordance to Aminot and Rey (2001), the basic structure of chl is retrapyrole macrocycle chelating a magnesium ion (Aminot and Rey 2001). During the chl degradation, chl molecule loses its magnesium ion and the resulting product of this process is pheophytin (Sanmartin et al. 2011; Matile et al. 1999). The chl degradation may occur under stress conditions, in which part of the chl *a* and chl *b* might have converted to phe *a* and phe *b* (Priyadarshini and Sujatha 2014). In addition, it is considered that chl *a* is more sensitive to pheopthytinization than chl *b* (Lichtenthaler 1987). The study showed that in plants exposed to Hg, the concentrations of phe *a* increased, indicating that Hg induced chl degradation. Our findings confirm thesis of other researchers who demonstrated that increased accumulation of phe was organism response to oxidative stress caused by heavy metals (Cr, Cd) and glyphosate (Rodriguez et al. 2007; Mobin and Khan 2007; Priyadarshini and Sujatha 2014; Gomes et al. 2016). The application of compost + CA as a soil amendments caused the limitation of chl *a* and *b* decrease due to activation of antioxidant system of *L. sativum* L.

Carotenoids are the non-enyzmatic antioxidants that can be synthetized during unfavorable environmental conditions. One of their roles is scavenging and deactivating free radicals (Safafar et al. 2015). Carotenoids include the following two classes: xanthophylls, which contain oxygen, and carotenes, which are purely hydrocarbons and contain no oxygen (Safafar et al. 2015). In this study, the investigations on total carotenoids (carotenes and xanthophylls) content in plants exposed to Hg and additional treatments were investigated. Based on the results, total carotenoid amount increased in plants exposed to Hg. These results stay in agreement with those presented by Anjum et al. (2014), who reported that carotenoid content in shoots of *Juncua maritimus* increased with increasing Hg treatment. According to Puzon et al. (2014) and Anjum et al. (2014), carotenoids were reported as a light-harvesting pigments and important quenchers of the singlet state of chlorophyll and singlet oxygen. The carotenoid content in leaves of *L. sativum* L. have also increased in plants cultivated in soil incorporated with compost + CA. Using compost + CA led to moderate increase of carotenoid content in relation to Hg treatments. The same tendency was observed by Ehsan et al. (2014) for *B. napus* L. plant treated with Cd and CA. Although the increase of total carotenoid content in plants exposed to Hg was observed, some further investigations should be provided to find out if this increase is related to activation of plant antioxidative system. Spectrophotometric method used for total carotenoid determination enables to estimate the only total and approximate amount of carotenoids, without detail description of the amount of individual carotenoids, like β-catorene, lycopene, zeaxanthin, and lutein (Jodłowska and Latała 2011).

Hg as a highly toxic contaminant affects the activity of plant enzymes. During this study, the POD activity was tested. The obtained results show the increase of POD activity in plants treated with Hg. This implies that *L. sativum* L. activated its enzymatic antioxidant system to survive in disadvantageous environmental conditions. The results are in line with findings of Chen et al. (2017) for rice treated with Hg. In accordance to Gao et al. (2012), POD can be considered to be one of the key enzymes that are involved in the removal of ROS because both its extracellular and intracellular forms participate in the breakdown of H_2_O_2_. An increase in POD activity is regarded as a reliable indicator of heavy metal impact because it is a response to increases in oxidative reactions, corresponding to an increase in peroxides, disruption of the cell membrane by lipid peroxidation, and free radicals produced from exposure to the phytotoxic fraction of accumulated metals (MacFarlane and Burchett 2001). An increased POD activity was also observed for Hg-treated plants with compost + CA application. This increase is correlated with increasing Hg accumulation in plant shoots. Moreover, also soil amendments, like compost and CA helped *L. sativum* L. to overcome the stress conditions. For instance, it has been reported that CA helped plants to overcome stress by enhancing their antioxidant enzyme activities under metal stress (Ehsan et al. 2014; Najeeb et al. 2009).

The recent study on Hg toxicity conducted by Chen et al. (2017) showed that Hg ions inhibited the photosynthetic pigments in rice seedlings. These authors had also investigated the influence of hydrogen sulfide (H_2_S) on the Hg toxicity in *O. sativa* L. According to results obtained in the study of Chen et al. (2017), H_2_S might act as an antioxidant to inhibit or scavenge ROS production, preventing at the same time oxidative damages. Use of sulfide effectively alleviated the Hg toxicity with an increase of photosynthetic pigment content in rice tissues and significantly promoted growth of rice seedlings (Chen et al. 2017).

*L. sativum* L. exposition to Hg limited metal accumulation in aboveground plant tissues. Low concentration of Hg in aerial tissues was also observed by other authors. Cargnelutti et al. (2006) showed that Hg concentration in shoots of *C. sativum* was relatively low. The comparative analysis of Hg accumulation showed slightly higher Hg concentration in stems of *L. sativum* L. than in leaves of plants cultivated in soil polluted by Hg. Similarly, results were obtained by Marrugo-Negrete et al. (2015) for *Jatropha curcas* in the first month of plant exposure to Hg.

Changing the soil substrate composition by combined use of compost + CA significantly increased Hg accumulation in both stems and leaves of *L. sativum* L. Increasing Hg concentration in these parts of plants may be a result of both the reduced Hg toxicity and increased Hg bioavailability in soil solution. The results of Smolinska (2015) showed that application of compost to the polluted soil may lead to formation of organic matter complexes with Hg, which in turn lead to decreasing Hg toxicity. On the other hand, CA enhanced other heavy metal accumulation (like Cd) because of its chelating ability (Ehsan et al. 2014). In accordance to Ehsan et al. (2014), increased heavy metal uptake in the presence of chelator, like citric acid, might be due to the organometallic complex formation in the soil solution. The formed organometallic complexes may break down at the root surface as a result of acid secretion, and then, free metal ion may be taken up by plant roots. This is the extracellular mechanism of heavy metal detoxication. Increasing Hg accumulation in plant shoots cultivated in soil supplemented by compost + CA can be also explained by activation of intracellular mechanisms of heavy metal detoxication. Metal chelates are produced in plant tissues from the metal ions with ligands like oxygen donor, sulfur donor, or nitrogen donor ligands taken up by plants from solution (Bibi et al. 2016). According to Bibi et al. (2016), divalent metal ions form highly stable complexes with carboxylic acid anions of terrestrial plants and carboxylate ions responsible for charge balancing within vacuoles of photosynthetic tissues. Moreover, the higher Hg accumulation in the presence of CA + compost may be explained by activation of antioxidant plant defense system, like increasing both carotenoids and POD activity in plants cultivated in those conditions.

Analysis of plant growth and response to Hg gave the information about the possibility of plant use for phytoextraction purposes. The main phytoextraction idea is to accumulate heavy metals from contaminated medium in aerial tissues of higher plants. Therefore, the capacity of plant to heavy metal accumulation in its shoots with reference to metal concentration in the soil (BCF) will help to assess the plant in terms of phytoextraction application. The BCF of *L. sativum* L. cultivated in Hg polluted soil was dependent on the Hg accumulation and its transfer to aerial tissues. The studies carried out by Su et al. (2007) and Su et al. (2008) showed that there are plant species, like Chinese brake fern (*Petris vittata*) that accumulate high concentrations of Hg in their shoots. The study conducted by Su et al. (2008) indicated that the highest Hg shoot accumulation occurred in fern grown in freshly contaminated soil; however, the aboveground tissue accumulation was dependent on the Hg concentration in soil and consisted 14–26% of total Hg accumulation by fern. Although the Hg accumulation by *L. sativum* L. was tested only in aboveground tissues, the previous findings showed that *L. sativum* L. translocated around 10% of total accumulated Hg into its shoots (Smolinska 2015). Soil enrichment by compost in soil/compost ratio 3/1 increased translocation of Hg to aerial plant tissues (Smolinska 2015). Almost half of the Hg accumulated by *L. sativum* L. was translocated into the shoots (Smolinska 2015). The comparative analysis of BCF for phytoextraction conducted on soil amended with compost (Smolinska 2015) and the soil amended with compost + CA shows that BCF increased after application of compost + CA. This suggests that the translocation of Hg to the aboveground parts of *L. sativum* L. was higher than obtained in the previous study of Smolinska (2015). Therefore, based on the results obtained from the study, *L. sativum* L. still can be considered as a Hg extractor specially during assisted phytoextraction.

The present study suggests that both changes in soil substrate properties as well as plant response to soil enhancements lead to increase Hg uptake and its translocation to aerial tissues of *L. sativum* L. The ideal plants for phytoextraction purposes are characterized by high ability of metal accumulation. In accordance to Sheoran et al. (2016), the hyperaccumulator is a plant that can accumulate metals and metalloid trace elements to a concentration that is 100 times greater than that of “normal” plants growing in the same environment. Until now, approximately 500 plant species from at least 45 plant families have been reported to hyperaccumulate various metals (Sheoran et al. 2016). However, no natural plant species with mercury hyperaccumulating properties have been yet identified (Su et al. 2007). Based on results obtained in the study, *L. sativum* L. cannot be classified to hyperaccumulator family. However, its high tolerance to Hg and short growing period create the possibility of using this plant in repeated phytoextraction (Smolinska and Szczodrowska 2016).

*L. sativum* L. is an edible plant and its use in phytoextraction process can be doubtful due to the risk of transferring pollutants into food chain. Nevertheless, the soils highly contaminated by heavy metals are excluded from agricultural use and activities are taken to monitor the state of pollution and the possible routes of its dissemination. Moreover, due to the absence of Hg hyperaccumulating plants, studies have been conducted on other species of consumer plants, like for example, parsley (*Petroselinum crispum*) and Indian mustard (*Brassica juncea*) (Bibi et al. 2016; Su et al. 2008). According to OECD, *L. sativum* L. can be used as a model plant for examining environmental stress (OECD 1984). Its short vegetation period and high sensitivity to even slight changes in environmental conditions have led to extensive research into its use in soil reclamation processes (Smolinska and Szczodrowska 2016). *L. sativum* L. has been tested not only for Hg phytoextraction but also for cadmium and nickel phytoremediation (Vakili and Aboutorab 2013; Mojiri et al. 2013). The high accumulation of heavy metals as well as low vegetation requirements of *L. sativum* L. confirm the chose of this plant specie in the context of its application to the biological remediation of heavy metal-contaminated soils (Vakili and Aboutorab 2013).

The results presented in this study are very promising. High translocation of Hg to aboveground tissues of *L. sativum* L. cultivated in soil enriched by simultaneous application of compost + CA is convinced of the suitability of using this plant specie together with the soil additives for reclamation processes. However, some other factors that affect Hg accumulation should be taken under consideration before field testing method. The plant factors like its biomass, good tolerance to high concentrations of metals in plant tissues, high translocation, and adaptability to specific sites are very important when choosing the appropriate plant species. On the other hand, equally important are the factors related to soil, like its type, moisture, pH, redox potential, cation exchange capacity, and biochemical processes (Sheoran et al. 2016). All of these factors can influence the success of assisted phytoextraction. Therefore, detail investigations should be provided before field testing method.

## Conclusion

*L. sativum* L. growth and development occurred despite the unfavorable growth condition caused by Hg presence in the soil. Hg accumulation in shoots of the plant was low but significantly increased after application of compost + CA into the soil due to both changing the soil substrate conditions as well as activation of plant protection mechanisms. The decreasing chl content in leaves of *L. sativum* L. under Hg exposition, related to its partial degradation to pheophytins, showed plant sensitivity to Hg ions. On the other hand, the changes of carotenoid content and increase of POD activity reveal that this plant is capable of tolerating Hg. The presented results have also shown that application of compost + CA as soil amendments increased plant resistance to Hg toxicity and at the same time increased Hg accumulation. This suggests that combined use of compost + CA may be helpful during assisted Hg phytoextraction.
